# Genome sequence of the *Medicago*-nodulating *Ensifer meliloti* commercial inoculant strain RRI128

**DOI:** 10.4056/sigs.4929626

**Published:** 2014-01-25

**Authors:** Wayne Reeve, Ross Ballard, Elizabeth Drew, Rui Tian, Lambert Bräu, Lynne Goodwin, Marcel Huntemann, James Han, Reddy Tatiparthi, Amy Chen, Konstantinos Mavrommatis, Victor Markowitz, Krishna Palaniappan, Natalia Ivanova, Amrita Pati, Tanja Woyke, Nikos Kyrpides

**Affiliations:** 1 Centre for Rhizobium Studies, Murdoch University, Western Australia, Australia; 2 South Australian Research and Development Institute, Urrbrae, South Australia, Australia; 3 School of Life and Environmental Sciences, Deakin University, Victoria, Australia; 4 Los Alamos National Laboratory, Bioscience Division, Los Alamos, New Mexico, USA; 5 DOE Joint Genome Institute, Walnut Creek, California, USA; 6 Biological Data Management and Technology Center, Lawrence Berkeley National Laboratory, Berkeley, California, USA

**Keywords:** root-nodule bacteria, nitrogen fixation, rhizobia, *Alphaproteobacteria*

## Abstract

*Ensifer meliloti* strain RRI128 is an aerobic, motile, Gram-negative, non-spore-forming rod. RRI128 was isolated from a nodule recovered from the roots of barrel medic (*Medicago truncatula*) grown in the greenhouse and inoculated with soil collected from Victoria, Australia. The strain is used in commercial inoculants in Australia. RRI128 nodulates and forms an effective symbiosis with a diverse range of lucerne cultivars (*Medicago sativa*) and several species of annual medic (*M. truncatula*, *Medicago littoralis* and *Medicago tornata*), but forms an ineffective symbiosis with *Medicago polymorpha*. Here we describe the features of *E. meliloti* strain RRI128, together with genome sequence information and annotation. The 6,900,273 bp draft genome is arranged into 156 scaffolds of 157 contigs, contains 6,683 protein-coding genes and 87 RNA-only encoding genes, and is one of 100 rhizobial genomes sequenced as part of the DOE Joint Genome Institute 2010 Genomic Encyclopedia for Bacteria and Archaea-Root Nodule Bacteria (GEBA-RNB) project.

## Introduction

*Ensifer meliloti* strain RRI128 is used in Australia to produce commercial peat cultures (referred to as Group AL inoculants) mainly for the inoculation of lucerne (*Medicago sativa* L.). Lucerne is sown on about 600, 000 ha annually (A. Humphries pers. com.) and is nearly always inoculated prior to sowing. RRI128 is also used for the inoculation of strand medic (*Medicago littoralis* Loisel) and disc medic (*Medicago tornata* (L.) Miller), a hybrid of the two former species, and bokhara clover (*Melilotus albus* Medik). RRI128 has been used commercially since 2000 when it replaced strain WSM826 [1]. Strain RRI128 was isolated from a nodule from the roots of barrel medic (*Medicago truncatula* Gaertn) growing in the greenhouse and inoculated with an alkaline sandy soil (pH_CaCl2_ 7.6) collected by J. Slattery, near Tempy, Victoria.

The strain was selected for use in commercial inoculants following assessment of its nitrogen fixation capacity (effectiveness), growth on acidified agar and saprophytic competence in an *in-situ* soil study [2], with supporting data of satisfactory performance at ten field sites. Additional testing has shown RRI128 to be effective on 28 cultivars of lucerne (Ballard unpub. data). It also forms effective symbiosis with a range of strand and disc medics [2] which show symbiotic affinity with lucerne [3,4].

Soil acidity has long been recognized as a constraint to lucerne nodulation [5] with some evidence that strains of *E. meliloti* have less acidity tolerance than *Ensifer medicae*, possibly due to their association with *Medicago* species that favor neutral to alkaline soils [6]. With RRI128, constraints to lucerne nodulation are observed around pH 5. Nodulation of lucerne seedlings inoculated with RRI128 was 42% at pH 5.0 in solution culture experiments [7] and observed to decline rapidly at field sites where pH_CaCl2_ was below 4.7 (Ballard, unpub. data). Other strains (e.g. SRDI672) have increased lucerne nodulation in solution culture at pH 4.8 (61% cf. 12% of lucerne seedlings with nodules) but are probably approaching the limit of acidity tolerance for *E. meliloti* [8].

Stable colony morphology and cell survival on seed make RRI128 amenable to commercial use. RRI128 produces colonies of consistent appearance and with moderate polysaccharide when grown on yeast mannitol agar, enabling easy visual assessment of culture purity. It differs in this regard from the strain it replaced (WSM826) which produced ‘dry’ and ‘mucoid’ colony variants, in common with many of the strains that nodulate lucerne and medic [9]. When applied correctly RRI128 has been shown to survive at more than 10,000 cells per lucerne seed at six weeks after inoculation [10]. Good survival may well be characteristic of *E. meliloti,* since former inoculant strain WSM826 is equally competent in this regard [11,12].

Here we present a preliminary description of the general features of *E. meliloti* strain RRI128 together with its genome sequence and annotation.

## Classification and general features

**Figure 1:**
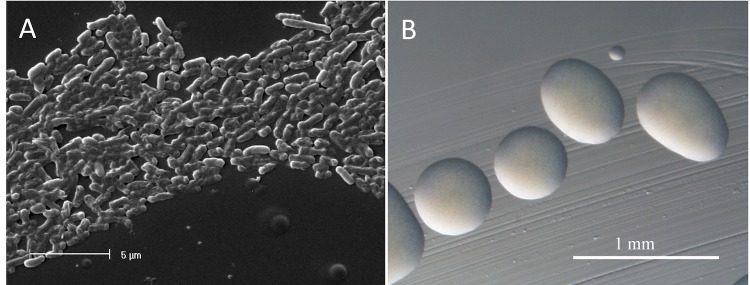
Images of *Ensifer meliloti* strain RRI128 using (A) scanning electron microscopy and (B) light microscopy to show the colony morphology on TY plates.

*Ensifer meliloti* strain RRI128 is a motile, non-sporulating, non-encapsulated, Gram-negative rod in the order *Rhizobiales* of the class *Alphaproteobacteria*. The rod-shaped form varies in size with dimensions of approximately 0.5 μm in width and 1.0-2.0 μm in length (Figure 1A). It is fast growing, forming colonies within 3-4 days when grown on TY [13] or half strength Lupin Agar (½LA) [14] at 28°C. Colonies on ½LA are opaque, slightly domed and moderately mucoid with smooth margins (Figure 1B).

**Figure 2:**
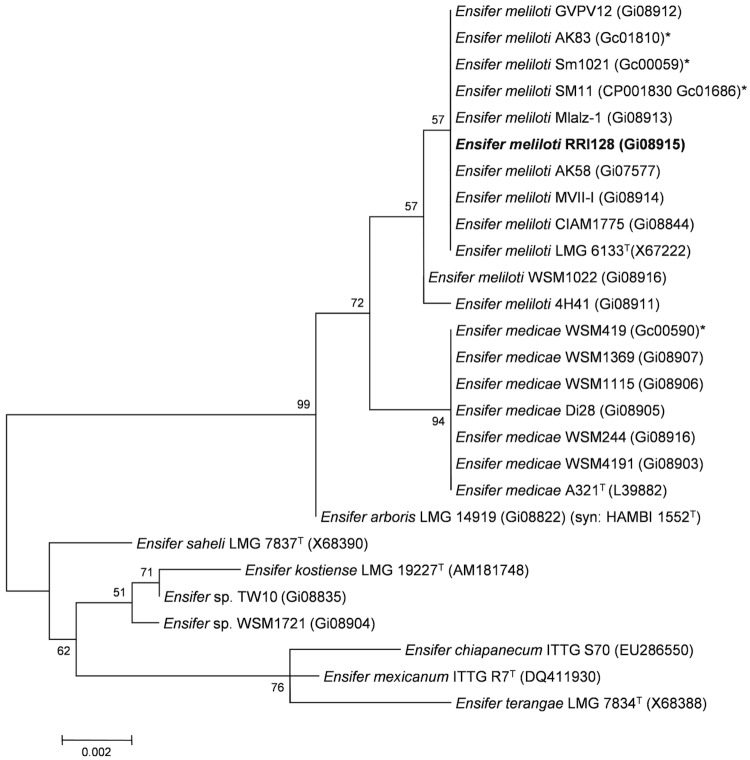
Phylogenetic tree showing the relationship of *Ensifer meliloti* strain RRI128 (shown in bold) with some of the root nodule bacteria in the order *Rhizobiales* based on aligned sequences of the 16S rRNA gene (1,307 bp internal region). All sites were informative and there were no gap-containing sites. Phylogenetic analyses were performed using MEGA [27], version 5.05. The tree was built using the maximum likelihood method with the General Time Reversible model. Bootstrap analysis [28] with 500 replicates was performed to assess the support of the clusters. Type strains are indicated with a superscript T. Brackets after the strain name contain a DNA database accession number and/or a GOLD ID (beginning with the prefix G) for a sequencing project registered in GOLD [29]. Published genomes are indicated with an asterisk.

Minimum Information about the Genome Sequence (MIGS) is provided in Table 1. Figure 2 shows the phylogenetic neighborhood of *Ensifer meliloti* strain RRI128 in a 16S rRNA gene sequence based tree. This strain has 100% sequence identity (1366/1366 bp) at the 16S rRNA sequence level to the fully sequenced *E. meliloti* Sm1021 [30] and 99% 16S rRNA sequence (1362/1366 bp) identity to the fully sequenced *E. medicae* strain WSM419 [31].

**Table 1:** **Classification and general features of *Ensifer meliloti* strain RRI128 according to the MIGS recommendations [**15,16**]**

**MIGS ID**	**Property**	**Term**	**Evidence code**
	Current classification	Domain *Bacteria*	TAS [15,16]
		Phylum *Proteobacteria*	TAS [17]
		Class *Alphaproteobacteria*	TAS [18]
		Order *Rhizobiales*	TAS [19]
		Family *Rhizobiaceae*	TAS [20]
		Genus *Ensifer*	TAS [21,22]
		Species *Ensifer meliloti*	TAS [23,24]
		Strain RRI128	
	Gram stain	Negative	IDA
	Cell shape	Rod	IDA
	Motility	Motile	IDA
	Sporulation	Non-sporulating	NAS
	Temperature range	Mesophile	NAS
	Optimum temperature	28°C	NAS
	Salinity	Non-halophile	NAS
MIGS-22	Oxygen requirement	Aerobic	IDA
	Carbon source	Varied	NAS
	Energy source	Chemoorganotroph	NAS
MIGS-6	Habitat	Soil, root nodule, on host	IDA
MIGS-15	Biotic relationship	Free living, symbiotic	IDA
MIGS-14	Pathogenicity	Non-pathogenic	NAS
	Biosafety level	1	TAS [25]
	Isolation	Root nodule	IDA
MIGS-4	Geographic location	Tempy, Vict., Australia	IDA
MIGS-5	Soil collection date	Circa 1995	IDA
MIGS-4.1 MIGS-4.2	Latitude Longitude	-35.1833 142.3833	IDA IDA
MIGS-4.3	Depth	0-10 cm	IDA
MIGS-4.4	Altitude	Not reported	

Evidence codes – IDA: Inferred from Direct Assay; TAS: Traceable Author Statement (i.e., a direct report exists in the literature); NAS: Non-traceable Author Statement (i.e., not directly observed for the living, isolated sample, but based on a generally accepted property for the species, or anecdotal evidence). These evidence codes are from the Gene Ontology project [26].

### Symbiotaxonomy

*Ensifer meliloti* strain RRI128 forms nodules on (Nod^+^) and fixes N_2_ (Fix^+^) with *Medicago sativa*, *Melillotus albus* and *Trigonella balansae* (Boiss. and Reuter). It also forms effective symbiosis with several species of annual medic (*M. truncatula*, *M. littoralis* and *M. tornata*) that happen to be closely related to each other based on their ability to be hybridized [5] and morphological and nucleotide sequence analyses of their relatedness [32]. RRI128 forms ineffective (white) nodules with *Medicago polymorpha,* a species that is generally recognized to have a more specific rhizobial requirement for effective symbiosis than *Medicago sativa* and *Medicago littoralis* [4,33] (Table 2).

**Table 2:** Compatibility of RRI128 with various *Medicago* and allied genera for nodulation (Nod) and N_2_-fixation (Fix).

**Species Name**	**Cultivar or line**	**Common Name**	**Growth Type**	**Nod**	**Fix**	**Reference**
*Medicago sativa*	*28 cultivars	Lucerne, Alfalfa	Perennial	+	+	[2]
*M. littoralis*	Harbinger, Herald, Angel	Strand medic	Annual	+	+	[2]
*M. tornata*	Tornafield, Rivoli	Disc medic	Annual	+	+	[2]
*M. tornata×littoralis*	Toreador	Hybrid disc medic	Annual	+	+	[2]
*M. truncatula*	Jester	Barrel medic	Annual	+	+	IDA
*M. polymorpha*	Scimitar	Burr medic	Annual	+(w)	-	IDA
*Trigonella balansae*	SA5045, SA32999, SA33025	Sickle fruited fenugreek	Annual	+	+	[34]
*Melilotus albus*	SA19917, SA35627, SA34665	Bokhara clover	Biennial	+	+	IDA

* 28 cultivars tested: Aquarius, Aurora, Cropper 9, Cuff 101, Eureka, Genesis, Hallmark, Hunterfield, Hunter River, Jinderra, ML 99, PL 55, PL 60, PL 69, Prime, SARDI Five, SARDI Seven, SARDI Ten, Sceptre, Sequel, Sequel-HR, Siriver, Trifecta, UQL1, Venus, WL525HQ, 54Q53 and 57Q75.

(w) indicates white nodules.

IDA: Inferred from Direct Assay; evidence code from the Gene Ontology project [26]

## Genome sequencing and annotation

### Genome project history

This organism was selected for sequencing on the basis of its environmental and agricultural relevance to issues in global carbon cycling, alternative energy production, and biogeochemical importance, and is part of the Community Sequencing Program at the U.S. Department of Energy, Joint Genome Institute (JGI) for projects of relevance to agency missions. The genome project is deposited in the Genomes OnLine Database [29] and an improved-high-quality-draft genome sequence in IMG/GEBA. Sequencing, finishing and annotation were performed by the JGI. A summary of the project information is shown in Table 3.

**Table 3:** Genome sequencing project information for *Ensifer meliloti* strain RRI128

**MIGS ID**	**Property**	**Term**
MIGS-31	Finishing quality	High-Quality-Draft
MIGS-28	Libraries used	1× Illumina Std library
MIGS-29	Sequencing platforms	Illumina HiSeq 2000
MIGS-31.2	Sequencing coverage	285× Illumina
MIGS-30	Assemblers	with Allpaths, version r39750, Velvet 1.1.04
MIGS-32	Gene calling methods	Prodigal 1.4
	Genbank ID	ATYP00000000
	Genbank Date of Release	September 5, 2013
	GOLD ID	Gi08915
	GenBank ID	X67222
	Database: IMG-GEBA	2513237091
	Project relevance	Symbiotic N_2_ fixation, agriculture

### Growth conditions and DNA isolation

*Ensifer meliloti* strain RRI128 was cultured to mid logarithmic phase in 60 ml of TY rich medium on a gyratory shaker at 28°C [35]. DNA was isolated from the cells using a CTAB (Cetyl trimethyl ammonium bromide) bacterial genomic DNA isolation method [36].

### Genome sequencing and assembly

The genome of *Ensifer meliloti* strain RRI128 was sequenced at the Joint Genome Institute (JGI) using Illumina [37] technology. An Illumina standard shotgun library was constructed and sequenced using the Illumina HiSeq 2000 platform, which generated 13,085,546 reads totaling 1,962 Mb of Illumina data.

All general aspects of library construction and sequencing performed at the JGI can be found at the JGI user home [36]. All raw Illumina sequence data was passed through DUK, a filtering program developed at JGI, which removes known Illumina sequencing and library preparation artifacts (Mingkun, L., Copeland, A. and Han, J., unpublished). The following steps were then performed for assembly: (1) filtered Illumina reads were assembled using Velvet [38], version 1.1.04, (2) 1–3 Kb simulated paired end reads were created from Velvet contigs using wgsim [39], (3) Illumina reads were assembled with simulated read pairs using Allpaths–LG [40] (version r39750).

**Parameters for assembly steps were:**
1. Velvet (Velvet optimizer params: --v --s 51 --e 71 --i 2 --t 1 --f "-shortPaired -fastq $FASTQ" --o "-ins_length 250 -min_contig_lgth 500")
2. wgsim (-e 0 -1 76 -2 76 -r 0 -R 0 -X 0,) (3) Allpaths–LG (PrepareAllpathsInputs:PHRED64=1 PLOIDY=1 FRAGCOVERAGE=125 JUMPCOVERAGE=25 LONGJUMPCOV=50, RunAllpath-sLG: THREADS=8 RUN=stdshredpairs TARGETS=standard VAPIWARNONLY=True OVERWRITE=True).

The final draft assembly contained 157 contigs in 156 scaffolds. The total size of the genome is 6.9 Mb and the final assembly is based on 1,962 Mb of Illumina data, which provides an average 285× coverage of the genome.

### Genome annotation

Genes were identified using Prodigal [41] as part of the Oak Ridge National Laboratory genome annotation pipeline. The predicted CDSs were translated and used to search the National Center for Biotechnology Information (NCBI) non-redundant database, UniProt, TIGRFam, Pfam, PRIAM, KEGG, COG, and InterPro databases. These data sources were combined to assert a product description for each predicted protein. Non-coding genes and miscellaneous features were predicted using tRNAscan-SE [42] RNAMMer [43], Rfam [44], TMHMM [45], and SignalP [46]. Additional gene prediction analyses and functional annotation were performed within the Integrated Microbial Genomes (IMG-ER) platform [47].

## Genome properties

The genome is 6,900,273 nucleotides with 61.98% GC content (Table 4) and comprised of 156 scaffolds (Figures 3a,3b,3c,3d,3e). From a total of 6,770 genes, 6,683 were protein encoding and 87 RNA only encoding genes. The majority of genes (78.79%) were assigned a putative function whilst the remaining genes were annotated as hypothetical. The distribution of genes into COGs functional categories is presented in Table 5.

**Table 4:** Genome Statistics for *Ensifer meliloti* strain RRI128

**Attribute**	**Value**	**% of Total**
Genome size (bp)	6,900,273	100.00
DNA coding region (bp)	5,931,611	85.96
DNA G+C content (bp)	4,276,906	61.98
Number of scaffolds	156	
Number of contigs	157	
Total gene	6,770	100.00
RNA genes	87	1.29
rRNA operons	1*	
Protein-coding genes	6,683	98.71
Genes with function prediction	5,334	78.79
Genes assigned to COGs	5,314	78.49
Genes assigned Pfam domains	5,505	81.31
Genes with signal peptides	569	8.40
Genes with transmembrane helices	1,483	21.91
CRISPR repeats	0	

*2 copies of 5S, 1 copy of 16S and 1 copy of 23S rRNA

**Figure 3a:**
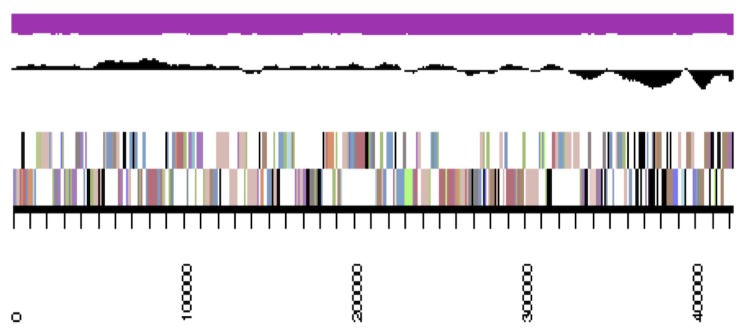
Graphical map of YU7DRAFT_scaffold_0.1 of the genome of *Ensifer meliloti* strain RRI128. From bottom to the top of each scaffold: Genes on forward strand (color by COG categories as denoted by the IMG platform), Genes on reverse strand (color by COG categories), RNA genes (tRNAs green, sRNAs red, other RNAs black), GC content, GC skew.

**Figure 3b:**
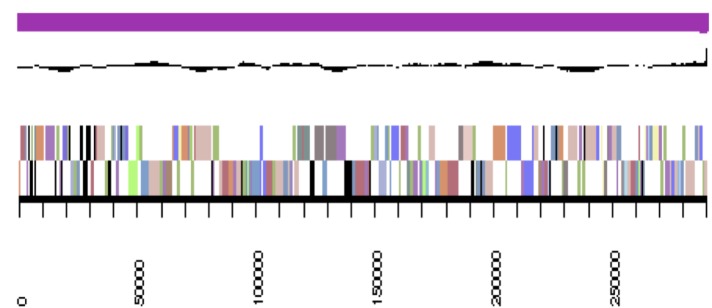
Graphical map of YU7DRAFT_scaffold_1.2 of the genome of *Ensifer meliloti* strain RRI128. From bottom to the top of each scaffold: Genes on forward strand (color by COG categories as denoted by the IMG platform), Genes on reverse strand (color by COG categories), RNA genes (tRNAs green, sRNAs red, other RNAs black), GC content, GC skew.

**Figure 3c:**
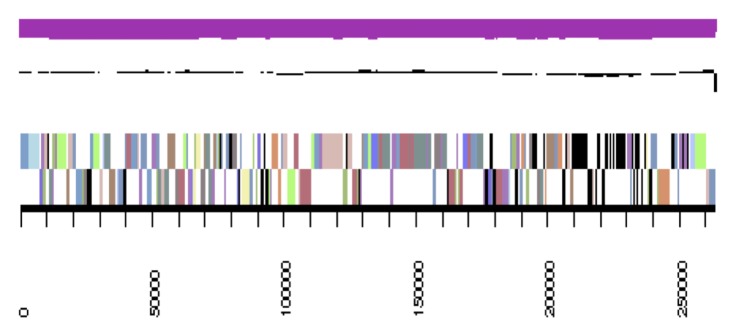
Graphical map of YU7DRAFT_scaffold_2.3 of the genome of *Ensifer meliloti* strain RRI128. From bottom to the top of each scaffold: Genes on forward strand (color by COG categories as denoted by the IMG platform), Genes on reverse strand (color by COG categories), RNA genes (tRNAs green, sRNAs red, other RNAs black), GC content, GC skew.

**Figure 3d:**
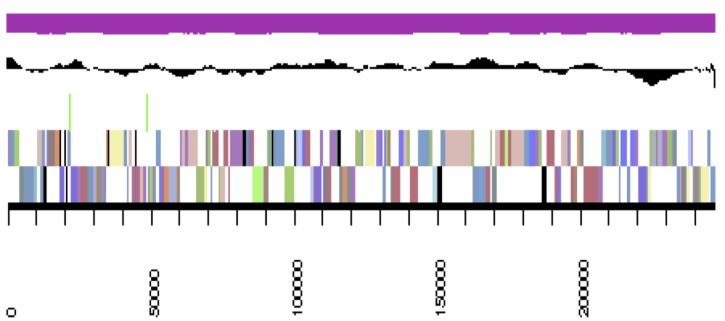
Graphical map of YU7DRAFT_scaffold_3.4 of the genome of *Ensifer meliloti* strain RRI128. From bottom to the top of each scaffold: Genes on forward strand (color by COG categories as denoted by the IMG platform), Genes on reverse strand (color by COG categories), RNA genes (tRNAs green, sRNAs red, other RNAs black), GC content, GC skew.

**Figure 3e:**
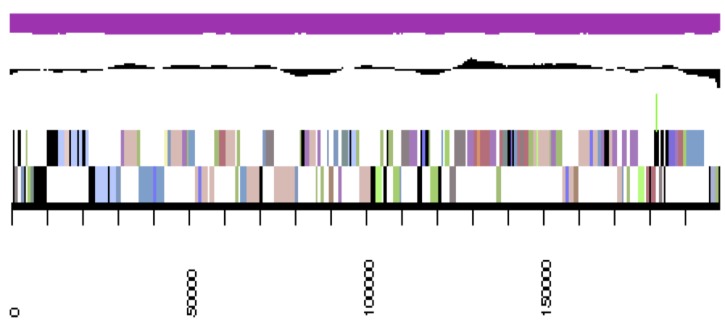
Graphical map of YU7DRAFT_scaffold_4.5 of the genome of *Ensifer meliloti* strain RRI128. From bottom to the top of each scaffold: Genes on forward strand (color by COG categories as denoted by the IMG platform), Genes on reverse strand (color by COG categories), RNA genes (tRNAs green, sRNAs red, other RNAs black), GC content, GC skew.

**Table 5:** Number of protein coding genes of *Ensifer meliloti* strain RRI128 associated with the general COG functional categories

C**ode**	**Value**	**%age**	**COG Category**
J	202	3.41	Translation, ribosomal structure and biogenesis
A	0	0.00	RNA processing and modification
K	520	8.78	Transcription
L	272	4.59	Replication, recombination and repair
B	2	0.03	Chromatin structure and dynamics
D	47	0.79	Cell cycle control, mitosis and meiosis
Y	0	0.00	Nuclear structure
V	61	1.03	Defense mechanisms
T	237	4.00	Signal transduction mechanisms
M	294	4.97	Cell wall/membrane biogenesis
N	75	1.27	Cell motility
Z	0	0.00	Cytoskeleton
W	1	0.02	Extracellular structures
U	116	1.96	Intracellular trafficking and secretion
O	186	3.14	Posttranslational modification, protein turnover, chaperones
C	355	6.00	Energy production conversion
G	594	10.03	Carbohydrate transport and metabolism
E	673	11.37	Amino acid transport metabolism
F	108	1.82	Nucleotide transport and metabolism
H	197	3.33	Coenzyme transport and metabolism
I	216	3.65	Lipid transport and metabolism
P	306	5.17	Inorganic ion transport and metabolism
Q	168	2.84	Secondary metabolite biosynthesis, transport and catabolism
R	705	11.91	General function prediction only
S	585	9.88	Function unknown
-	1,456	21.51	Not in COGS
